# Differentiating Black and Hispanic: outcome differences of segregated communities and police shootings in the USA, 2015–2020

**DOI:** 10.1186/s40621-022-00372-y

**Published:** 2022-03-03

**Authors:** Timothy F. Leslie, Cara L. Frankenfeld, Angela J. Hattery

**Affiliations:** 1 Department of Geography and Geoinformation Science, George Mason University, 4400 University Dr., MS 6C3, Fairfax, VA 22030 USA; 2 Public Health Program, University of Puget Sound, 1500 N. Warner St., Tacoma, WA 98416 USA; 3 Department of Women and Gender Studies, University of Delaware, 25 N. College Ave., Newark, DE 19716 USA

**Keywords:** Segregation, Police, Shootings, Racial

## Abstract

**Background:**

Police shootings are unevenly spatially distributed, with substantive spikes throughout the USA. While minorities are disproportionately the victims of police force, social or structural factors associated with this distribution are not well understood. The objective of this work was to evaluate police shootings in relation to victim race or ethnicity and residential segregation and racial diversity.

**Methods:**

Validated crowdsourced data from the Washington Post’s Fatal Force (2015–2020) were linked with census tract-level data from the American Community Survey. Residential minority dissimilarity, interaction, and a racial and ethnic diversity metric were calculated in order to assess the potentially variant importance of evenness in distribution, exposure likelihood, and general representation. Logistic and multinomial regression was used to model associations between segregation and diversity, adjusted for other ecological characteristics. Analyses were stratified by victim race or ethnicity (Black, Asian, or Hispanic).

**Results:**

Across all races combined, the odds of a police shooting in a particular census tract were associated with non-Hispanic Black dissimilarity (OR = 0.98, 95% CI 0.97, 0.99) and racial and ethnic diversity (1.046, 95% CI 1.044, 1.060). Areas with higher racial diversity had a higher likelihood of having a police shooting event with Black victims (OR = 1.092, 95% CI 1.064, 1.120) or Asian victims (OR = 1.188, 1.051, 1.343) than less diverse areas. Higher non-Hispanic Black interaction was associated with a lower likelihood of having a police shooting event with Black victims (OR = 0.914, 95% CI 0.833, 0.946) than lower interaction areas. Higher Hispanic dissimilarity was associated with a lower likelihood of having a police shooting event with a Hispanic victim (OR = 0.398, 95% CI 0.324, 0.489) than lower dissimilarity areas.

**Conclusions:**

The variant effects of residential segregation are only seen when victims are analyzed separately by race. There appears to be a protective effect for Hispanic populations in Hispanic communities, while the reverse is true of Black individuals. We urge law enforcement responsible for locations with segregated communities to monitor individual interactions that police have with residents as well as the patterns of frequency and context of those interactions.

## Background

The US death by firearm assault rate is more than 10 times higher than the combined number of deaths for the next 4 highest countries by Gross Domestic Product combined (Marczak et al. 2016). Police shootings, in particular, are being regarded as a public health issue due to their increase in occurrence over the past 30 years, as well as a continued disparity in racialized occurrences (GBD 2021). The probability of being fatally shot while Black and unarmed in the USA is 3.49 times more likely than if we are White and unarmed (Ross 2015). Injuries due to police intervention have been found to be 4.9 times higher for Black individuals^1^ compared to White individuals (Feldman et al. 2019). These differences remained despite increasing public scrutiny and growing voices for the concern over the determinants of the inequities or their potential solutions.

Characteristics of the environment have long been understood to influence perceptions and behavior during a police encounter (Kania and Mackey 1977; Fyfe 1980, 1988). Hemenway et al. (2019) found that states with higher household gun ownership prevalence have higher rates of police shootings. Kivisto (2017) found that stricter firearm legislation was associated with lower rates of fatal police shootings. Communities with higher population of minorities experience more fatal police shootings if the population has a higher number of Black residents, although predominantly Hispanic areas did not have the same trends (Snyder 2013). Fatal police shooting events are more likely to occur in large metropolitan counties with low median incomes, higher proportion of Black residents, and higher levels of financial inequality (Ross 2015).

In addition to differing prevalences of demographic and socioeconomic factors, the contrast and separation between communities—termed residential segregation—are an additional factor in police behavior (Wright et al. 2021). At the state level, increases in a racism index were found to be a significant driver in the disparity between fatal police shootings of unarmed White and Black individuals, with the association being driven primarily by the residential segregation component of the index (Mesic et al. 2018). These social forces may be mediated through social segregation resulting from the separation of populations along different sociodemographic axes, including race, which can occur through self-selection (Bishop 2009), but may be more determined by structural factors. Structural factors include racial segregation in housing that resulted in segregated neighborhoods and produced a variety of negative outcomes for Blacks in the USA (Massey et al. 1998). Blacks with income levels corresponding to middle class are significantly less likely to live in middle-class neighborhoods than their White counterparts, but instead are more likely to live in low-income neighborhoods, which are characterized by higher crime rates, lower resourced schools, and fewer economic opportunities. Conversely, Whites are far more likely to live in neighborhoods that are both racially and economically homogenous and have better resourced schools, more economic opportunities, and lower crime (O’Hanlon 2017; Reardon et al. 2015). Research focused on racial segregation in Alabama and Mississippi also revealed that the impact of residential segregation was far more profound for Blacks than for Whites: Whites in counties with a higher proportion of Black individuals did not experience lower well-being than Whites in counties with a lower proportion of Black individuals, but the converse was true for Blacks as they, on average, experienced higher levels of well-being when they did not live in majority Black counties (Hattery and Smith 2007).

Segregation has been linked in disparities in mortality in sum and across a number of chronic illnesses (Frankenfeld et al. 2022), including heart disease (Williams and Collins 2001), cancer diagnosis and treatment (Dai 2010; Menon et al. 2020), diabetes (Gittner et al. 2017), infant mortality (Hattery and Smith 2007), incidence or prevalence of drug overdose deaths (Frankenfeld and Leslie 2019), and domestic violence (Miles-Doan 1998). Some types of segregation have been linked to increased assaults (Krieger et al. 2017), school bullying (Fu et al. 2013), homicides (Frankenfeld and Leslie 2021), and firearms violence (Beard et al. 2017) in a region. As a result of structural racism and legally enforced segregation practices like redlining and housing covenants, as well as personal bias among realtors and home sellers, many communities in the USA from the block to the county remain highly segregated by race (Massey 1990, 2015; Sampson and Sharkey 2008; Jacoby et al. 2018; Arcaya et al. 2018).

Many police departments that are in the news for cases of police killings or police brutality have a force that had a substantially different racial makeup compared to the population they policed (Jetelina et al. 2017). Racial bias in neighborhood police encounters has been documented as a cause for action by scholars in a number of social science fields (Hall et al. 2016; Gilbert and Ray 2016; Vitale 2017). Concerns over police violence have reached the point that government documents such as driving manuals now provide instructional guidance on what drivers should do during traffic stops to reduce the likelihood of being shot (ADOT 2018), while calls to “defund the police” have led to serious inquiry into the breadth of service that police officers undertake and other options that may be employed that limit or constrain traditional police response (Lum et al. 2021; Jacobs et al. 2021). In addition, the importance of understanding the relationship between race and police shootings has been a consistent source of scholarly inquiry (Fyfe 1982; Hemenway et al. 2019). State-level analysis of residential segregation and police shootings has found a strong effect in the Black–White disparity in firearm homicide rate (Knopov et al. 2019). While shootings across the USA have been examined as an occurrence (Ross 2015), our aim is to further the ongoing narrative both with and within police departments with regards to community race relations.

Segregation can be measured and calculated in a number of different ways (Massey et al. 1998), and each of the ways yields different information about the nature and magnitude of segregation. We utilize multiple residential segregation measures to evaluate associations with population outcomes to capture each of these particular outcomes. Racialized decision-making could be expressed more strongly in places that have uneven population distributions of a particular minority. Within the scholarship of racial inquiry and police behavior, we build upon the commonly used metric of dissimilarity, which examines the evenness or variance in minority population distribution and has been used elsewhere (e.g., Siegel et al. 2021’s application focused on Black populations). Alternatively, racist behavior might be derived by the expectations of a racial majority meeting a racial minority. We operationalize this through the metric of interaction, a measure of the likelihood of a majority member encountering a minority member. Finally, uneven outcomes could be a function of the relative ethnic diversity (or lack thereof) within a region. We implement a group-based metric in order to assess the importance of the presence of a wider number of minority types. Additionally, we consider Hispanic and Asian populations in both our “victims” and “segregation” calculations with the aim to identify whether there are particular elements of segregation, whether in measurement or racial specificity, that are connected to higher rates of fatal police shootings. This breadth provides a broader understanding of the potential mechanisms by which the geographic environment may be an important contributor to population health, including the occurrence racial and ethnic differences in police shootings.

## Methods

### Fatal force data

The Washington Post logs fatal shootings by on-duty police officers in the USA and makes the data publicly accessible as the Fatal Force database (Washington Post 2021). The database utilizes news accounts, social media postings, and police reports as sources of information (Washington Post 2016) and has been found to be consistent with other open-source police shootings databases (Comer and Ingram 2022). Data have been collected and reported from 2015, and, for this analysis, data from January 2015 through December 2020 were utilized. Data were geocoded to census tracts using the latitude and longitude information provided in the database. In the Fatal Force Data, race and ethnicity are classified in a single variable (White, Black, Asian, Hispanic, Native American, or other). Given that Hispanic often supersedes race in this categorization, the presumption is that White, Black, and Asian are non-Hispanic groups, and we use non-Hispanic classifications in the geographic data (described in the next section). However, when referring to the victims, we use the classifications as stated in the data, and the number of fatal shooting events in each census tract was calculated by for race and ethnic groups: Hispanic, White, Black, Asian, and other.

### US population and geographic characteristics data

Data about the US population overall and by census tracts were obtained from the US Census for relevant characteristics. For overall population and populations by year, resident population data were obtained from the Monthly Population Estimates for the USA: April 1, 2010, to December 1, 2020 (table: NA-EST2019-01). Estimates for July 1 for the years 2015–2020 were used for each year, and the average across these values was used for the overall population. For age and gender groups, stratified data were obtained from the Annual Estimates of the Resident Population for Selected Age Groups by Sex for the USA: April 1, 2010, to July 1, 2019 (table: NC-EST2019-AGESEX). Data for resident population were aggregated by age-groups (5–19, 20–34, 35–49, and 50+ years) and gender (male and female) and averaged across years 2010 to 2019. Race and ethnicity resident population data were obtained from the Annual Estimates of the Resident Population by Sex, Race, and Hispanic Origin for the USA: April 1, 2010, to July 1, 2019 (table: NC-EST2019-SR11H). Census data were aggregated by race and ethnicity (Hispanic, non-Hispanic White, non-Hispanic Black, non-Hispanic Asian, non-Hispanic Native American, and non-Hispanic Other).

### Segregation calculations

In order to calculate residential segregation characteristics by race and ethnicity for census tracts, block group-level American Community Survey five-year estimates data (2015–2019) were obtained (table B03002). We follow Massey and Denton (1988) as well as Oka and Wong (2014) in our residential segregation metric selection. For residential segregation, dissimilarity and interaction were used in this analysis. Non-Hispanic White population was used as the majority population for the calculations. While in some areas, the majority may not be non-Hispanic White individuals, the calculations account for this in the use of absolute values. We incorporate multiple segregation metrics as they capture distinct elements (Harris and Johnston 2018). Dissimilarity provides a measure of evenness, and interaction is a measure of exposure that looks at the likelihood the minority and majority groups occupy the same area. The variables included in the calculations are the minority population of area i (x_i_), the total population of area i (t_i_), the ratio of x_i_ to t_i_ (p_i_), the majority population of area i (y_i_), the sum of all x_i_ (X), the sum of all t_i_ (T), and the ratio of X to T (P). Segregation measures were calculated for Hispanic, Black, and Asian as minority populations. The theoretical range for these values is from 0 to 1, with 1 indicating complete dissimilarity or complete interaction.

The interaction calculation

$$\mathop \sum \limits_{i = 1}^{n} \left[ {\left( {\frac{{x_{i} }}{X}} \right)\left( {\frac{{y_{i} }}{{t_{i} }}} \right)} \right]$$

The dissimilarity calculation

$$\frac{{\mathop \sum \nolimits_{i = 1}^{n} \left[ {t_{i} \left| {\left( {p_{i} - P} \right)} \right|} \right]}}{{\left[ {2TP\left( {1 - P} \right)} \right]}}$$

Diversity is a composition characteristic of the geographic unit and was calculated as the negative of the summation of the percentage of mutually exclusive groups multiplied by its natural logarithm. The mutually exclusive groups for racial diversity were non-Hispanic White, non-Hispanic Black, non-Hispanic American Indian or Alaska Native, non-Hispanic Asian, non-Hispanic Native Hawaii or Pacific Islander, non-Hispanic other, and Hispanic. This measure incorporates the effect of multiple minority groups rather than focusing on measures specific to a particular group. The theoretical range for this value is 0 to 1, with 1 indicating the highest diversity.

There are no agreed-upon geographic characteristic covariates for segregation analyses, and we used the Social Vulnerability Index (SVI) as a guide for relevant geographic-level social characteristics. The Agency for Toxic Substances & Disease Registry SVI is a tool created to identify and map communities that will most likely need support before, during, and after a hazardous event (Flanagan et al. 2011). Census tract-level data were downloaded for SVI 2018 (utilizing American Community Survey 5-year estimates from 2014 to 2018), and values for SVI themes were used as covariates in this analysis. SVI themes include socioeconomic status; household composition and disability; minority status and language; and housing type and transportation. Socioeconomic status theme incorporates data about percentage below poverty, percentage unemployed, percentage with no high school diploma, and mean income. Household composition and disability theme incorporates data about percentage individuals of age 65 and older and age 17 or younger, percentage of population older than age 5 with a disability, and percentage of single-parent households. Minority status and language theme incorporates data about percentage of minority individuals and percentage that speak English “less than well.” Housing type and transportation theme incorporates information about the percentages of multi-unit structures, mobile homes, crowded housing, households with no vehicle, and group quarters.

### Statistical analysis

Frequencies and percentages of fatal police shootings were calculated to describe the population of all shootings in the database from 2015 through 2020 (*n* = 6224). Frequencies were used to calculate incidence per 100,000 population using the corresponding relevant population. There were 5183 events that had both locations that could be geocoded to census tract (*n* = 5923) as well as had detail on the racial characteristics of the victim (*n* = 5674) in order to be included in the analysis to evaluate the associations with geographic characteristics. An examination of the removed fields (events unable to be geocoded) found no identifiable bias with regards to race or other characteristics of the victim.

Segregation, diversity, and SVI themes in raw form are scaled from 0 to 1 and were transformed to 0 to 10 scale for analysis by multiplying the values by 10. Pearson correlations were used to evaluate for potential collinearity between measures of segregation and other social geographic characteristics, and in the absence of strong correlations (Table 1), it was appropriate to include SVI themes as covariates for segregation and diversity. There were few instances of more than two events occurring in a census tract for each race or Hispanic ethnicity, and each census tract was classified as having 0, 1, 2+ events based on race or ethnicity of the victim for White, Black, Asian, and Hispanic. There was an insufficient frequency of events for Native American or other for those groups to be analyzed. Analyses were conducted at the census tract level. Multinominal logistic regression was used to model the odds of event for 1 and 2+ versus 0 events for diversity and each segregation measure. Analyses were also collapsed to evaluate the odds of any event (1+ versus 0) in relation to diversity and each segregation measure, and logistic regression was used for the analysis. Analyses were adjusted for SVI themes and log total population of the census tract. In order to consider possible differential effects by type of segregation, there are a number of comparisons made and used in statistical hypothesis testing. Due to the debated issues with applying multiple comparisons testing (Perneger 1998; Nakagawa 2004; Gelman et al. 2012), such measures have not been applied. However, aligned with recommendations from others (Nakagawa 2004), effect estimates and confidence intervals have been provided so that readers consider this issue in their interpretations of the results. Analyses were conducted using Stata (version 15, StataCorp, USA).

**Table 1:** Pearson correlation coefficients for relationships between segregation, diversity, and Social Vulnerability Index (SVI) themes

Geographic characteristic	Dissimilarity, non-Hispanic Black	Dissimilarity, non-Hispanic Asian	Dissimilarity, Hispanic	Interaction, non-Hispanic Black	Interaction, non-Hispanic Asian	Interaction, Hispanic	Racial and ethnic diversity	SVI, Socioeconomic Status	SVI, Household Composition and Disability	SVI, Minority Status and Language	SVI, Housing Type and Transportation
Dissimilarity, non-Hispanic Black	1.00										
Dissimilarity, non-Hispanic Asian	0.20	1.00									
Dissimilarity, Hispanic	0.13	0.24	1.00								
Interaction, non-Hispanic Black	− 0.30	− 0.07	− 0.08	1.00							
Interaction, non-Hispanic Asian	− 0.04	− 0.38	− 0.11	0.33	1.00						
Interaction, Hispanic	0.00	− 0.05	− 0.23	0.42	0.38	1.00					
Diversity, Racial and ethnic	− 0.35	− 0.33	− 0.16	− 0.05	− 0.02	− 0.14	1.00				
SVI, socioeconomic status	0.02	0.29	0.20	− 0.22	− 0.29	− 0.28	0.11	1.00			
SVI, household composition and disability	0.08	0.32	0.18	-0.11	-0.18	-0.12	-0.14	0.57	1.00		
SVI, minority status and language	-0.12	-0.12	-0.05	-0.26	-0.21	-0.35	0.62	0.41	0.00	1.00	
SVI, housing type and transportation	-0.09	0.05	0.03	-0.15	-0.17	-0.22	0.19	0.52	0.27	0.32	1.00

## Results

There were 5949 fatal shootings involving police officers documented in the database from January 2015 through December 2020 (Table 2). The overall annual incidence of fatal shootings was 0.31 per 100,000 population. Annual incidence of approximately 1000 was similar across the years included. Fatal shootings were more common in Mountain, East and West South Central, and Pacific Census regions, and less common in New England and Middle Atlantic. Incidence was highest in young adults and males. Higher incidence was observed in Black and Native American residents than other race and ethnic groups.

**Table 2:** Characteristics of fatal police shootings and annual incidence for selected population groups from 2015 to 2020

Characteristics	n	%	US Census Population Estimates*	per 100,000 population
All, 2015–2020	5949	100.0	325,561,090	0.305
*Year*				
2015	993	16.7	320,635,163	0.310
2016	960	16.1	322,941,311	0.297
2017	986	16.6	324,985,539	0.303
2018	990	16.6	326,687,501	0.303
2019	999	16.8	328,239,523	0.304
2020	1021	17.2	329,877,505	0.310
*Region*				
New England	112	1.9	14,790,786	0.126
Middle Atlantic	299	5.0	41,242,349	0.121
South Atlantic	1138	19.1	64,531,706	0.294
East South Central	441	7.4	19,019,070	0.386
West South Central	909	15.3	39,857,459	0.380
East North Central	585	9.8	46,847,718	0.208
West North Central	361	6.1	21,260,389	0.283
Mountain	883	14.8	24,171,762	0.609
Pacific	1221	20.5	52,976,569	0.384
*Age*				
6–19	299	5.0	62,137,807	0.080
20–34	2486	41.8	67,216,130	0.616
35–49	1878	31.6	61,642,990	0.508
50–91	1038	17.4	113,886,790	0.152
Unknown	248	4.2		
*Gender*				
Male	5688	95.6	159,893,431	0.593
Female	260	4.4	164,804,376	0.026
Unknown	1	0.0		
*Race and ethnicity*				
White	2774	46.6	197,655,170	0.234
Black	1444	24.3	40,534,100	0.594
Asian	103	1.7	18,109,812	0.095
Native American	84	1.4	2,403,978	0.582
Hispanic	1018	17.1	58,498,253	0.290
Other	47	0.8	7,496,494	0.104
Unknown	479	8.1		
*Manner of death*				
Shot	5649	95.0		
Shot and Tasered	300	5.0		
*Threat*				
Attack	3866	65.0		
Other	1891	31.8		
Undetermined	192	3.2		
*Signs of mental illness*				
No	4555	76.6		
Yes	1394	23.4		

*Estimates for all and by year represent 2015–2020; estimates for region, age, gender, race, and ethnicity are averages of 2015–2019 estimates based on currently available data

In relation to events regardless of race or ethnicity of the victim, non-Hispanic Black dissimilarity was inversely associated with an event in that census tract and racial and ethnic diversity was positively associated with an event occurring in the tract (Table 3). For White victims, dissimilarity for any type (non-Hispanic Black, Hispanic, or non-Hispanic Asian) was inversely associated, and interaction of any type and diversity was positively associated, with an event involving a White victim occurring in the census tract. For Black victims, Hispanic and non-Hispanic Asian dissimilarity and diversity were positively associated and interaction of any type was inversely associated with an event involving a Black victim occurring in the tract. For Hispanic victims, Hispanic and non-Hispanic Asian dissimilarity and diversity were inversely associated and non-Hispanic Asian interaction was positively associated with an event involving a Hispanic victim occurring in the tract. For Asian victims, Hispanic and non-Hispanic Asian dissimilarity was inversely associated and diversity was positively associated with an event involving an Asian victim occurring in the tract. There was some evidence of stronger associations when events were disaggregated from any to 1 and 2+ for any race or ethnicity victim and diversity, non-Hispanic Asian interaction and diversity for White victims, Hispanic dissimilarity and interaction for Black victims, and Hispanic dissimilarity and diversity for Hispanic victims.

**Table 3:** Association between any event in a census tract for measures of segregation and diversity by race and Hispanic ethnicity, adjusted for social vulnerability index themes and log population

Group	N tracts	Dissimilarity, non-Hispanic Black		Dissimilarity, Hispanic		Dissimilarity, non-Hispanic Asian		Interaction, non-Hispanic Black		Interaction, Hispanic		Interaction, non-Hispanic Asian		Diversity, race, and ethnic	
		OR	95% CI	OR	95% CI	OR	95% CI	OR	95% CI	OR	95% CI	OR	95% CI	OR	95% CI
*All*															
0	68,563	1.000	Ref	1.000	Ref	1.000	Ref	1.000	Ref	1.000	Ref	1.000	Ref	1.000	Ref
1+	4766	0.984^†^	0.972, 0.996	0.998	0.981, 1.016	0.999	0.991, 1.007	1.003	0.989, 1.016	1.002	0.987, 1.017	1.006	0.993, 1.019	1.042^†^	1.029, 1.056
2+	417	0.972	0.930, 1.016	0.977	0.913, 1.046	0.981	0.954, 1.008	1.016	0.966, 1.067	1.012	0.957, 1.071	1.037	0.991, 1.086	1.109^†^	1.060, 1.160
Any (1+)	5183	0.983^†^	0.972, 0.994	0.997	0.980, 1.014	0.998	0.990, 1.006	1.004	0.991, 1.017	1.003	0.988, 1.018	1.008	0.995, 1.021	1.046^†^	1.033, 1.060
*White*															
0	71,224	1.000	Ref	1.000	Ref	1.000	Ref	1.000	Ref	1.000	Ref	1.000	Ref	1.000	Ref
1	2410	0.978^†^	0.963, 0.994	0.946^†^	0.921, 0.973	0.979^†^	0.968, 0.990	1.040^†^	1.024, 1.056	1.047^†^	1.029, 1.066	1.029^†^	1.013, 1.045	1.103^†^	1.082, 1.125
2+	112	0.966	0.891, 1.047	0.924	0.801, 1.065	1.024	0.976, 1.075	1.070	0.995, 1.151	1.038	0.954, 1.131	1.081^†^	1.010, 1.157	1.318^†^	1.191, 1.458
Any (1+)	2522	0.978^†^	0.963, 0.993	0.945^†^	0.920, 0.971	0.981^†^	0.970, 0.992	1.041^†^	1.025, 1.057	1.047^†^	1.029, 1.066	1.031^†^	1.015, 1.047	1.111^†^	1.090, 1.132
*Black*															
0	72,428	1.000	Ref	1.000	Ref	1.000	Ref	1.000	Ref	1.000	Ref	1.000	Ref	1.000	Ref
1	1253	0.981	0.959, 1.004	1.142^†^	1.116, 1.169	1.048^†^	1.034, 1.063	0.923^†^	0.894, 0.954	0.907^†^	0.876, 0.940	0.939^†^	0.910, 0.969	1.082^†^	1.056, 1.109
2 +	65	0.997	0.898, 1.106	1.223^†^	1.110, 1.347	1.027	0.963, 1.095	0.935	0.802, 1.090	0.760^†^	0.615, 0.940	0.917	0.786, 1.069	1.056	0.950, 1.174
Any (1 +)	1318	0.982	0.960, 1.004	1.145^†^	1.120, 1.172	1.047^†^	1.033, 1.062	0.924^†^	0.895, 0.954	0.902^†^	0.871, 0.934	0.938^†^	0.910, 0.967	1.081^†^	1.056, 1.107
*Hispanic*															
0	72,810	1.000	Ref	1.000	Ref	1.000	Ref	1.000	Ref	1.000	Ref	1.000	Ref	1.000	Ref
1	883	0.988	0.959, 1.017	0.406^†^	0.333, 0.495	0.966^†^	0.945, 0.986	0.977	0.933, 1.022	1.042	0.991, 1.095	1.048^†^	1.007, 1.090	0.970^†^	0.945, 0.995
2+	53	0.859	0.695, 1.060	0.159^†^	0.049, 0.519	0.966	0.886, 1.053	1.110	0.906, 1.360	1.126	0.886, 1.431	1.053	0.867, 1.279	0.907^†^	0.824, 0.998
Any (1 +)	936	0.983	0.955, 1.012	0.391^†^	0.321, 0.476	0.966^†^	0.946, 0.986	0.982	0.939, 1.026	1.046	0.996, 1.098	1.048^†^	1.008, 1.090	0.965^†^	0.942, 0.990
Asian*															
0	73,649	1.000	Ref	1.000	Ref	1.000	Ref	1.000	Ref	1.000	Ref	1.000	Ref	1.000	Ref
Any (1+)	97	1.053	0.981, 1.130	0.473^†^	0.257, 0.870	0.913^†^	0.842, 0.991	0.990	0.889, 1.102	0.985	0.869, 1.118	1.015	0.915, 1.125	1.211^†^	1.075, 1.364

*There were too few instances of more than 2+ events for Asian victims to disaggregate to 1 and 2+ for Asian population group

^†^*p* < 0.05

Dissimilarity, interaction, and diversity had independent associations with events when mutually adjusted (Table 4). For Black victims, non-Hispanic Black interaction was inversely associated and diversity was positively associated with an event in the tract. For Hispanic victims, Hispanic dissimilarity was inversely associated with an event in the tract. For Asian victims, diversity was positively associated with an event in the tract. For both Black and Hispanic victims, all SVI themes were positively associated with an event occurring in the tract. For Asian victims, minority status and language and housing type and transportation SVI themes were positively associated with an event occurring in the tract.

**Table 4:** Association between any event in a census tract for measures of segregation, diversity, and social vulnerability by race and Hispanic ethnicity

Geographic characteristic*	Non-Hispanic Black		Hispanic		Non-Hispanic Asian	
	OR	95% CI	OR	95% CI	OR	95% CI
Dissimilarity	0.993	0.968, 1.019	0.398^†^	0.324, 0.489	0.936	0.857, 1.022
Interaction	0.914^†^	0.883, 0.946	1.004	0.956, 1.056	0.988	0.884, 1.104
Diversity	1.092^†^	1.064, 1.120	0.992	0.966, 1.019	1.188^†^	1.051, 1.343
SVI, socioeconomic status	1.196^†^	1.157, 1.236	1.098^†^	1.054, 1.143	0.926	0.828, 1.036
SVI, household composition and disability	1.055^†^	1.030, 1.081	1.047^†^	1.017, 1.077	1.048	0.961, 1.143
SVI, minority status and language	1.041^†^	1.012, 1.070	1.393^†^	1.336, 1.452	1.180^†^	1.042, 1.336
SVI, housing type and transportation	1.029^†^	1.005, 1.054	1.048^†^	1.019, 1.079	1.104^†^	1.014, 1.201
Log population	1.076^†^	0.957, 1.209	2.073^†^	1.791, 2.400	1.052	0.680, 1.628

*Models mutually adjusted for characteristics in the table. Dissimilarity corresponds to the population group, e.g., non-Hispanic Black dissimilarity and interaction for models that evaluate Black victim events

^†^*p* < 0.05

## Discussion

Our results evidence an association with racial residential segregation on policing behavior beyond correlations with other tract socioeconomic characteristics. Explanations for this policing difference have been a consistent topic of interdisciplinary scholarship. The overpolicing of minority communities has been well documented (Perry 2006; Weitzer 2017). For example, racial residential segregation and the subsequent effects on police shootings are consistent with research on differential applications of racial profiling biases in campaigns such as “stop and frisk” (Hattery and Smith 2021). Furthermore, when Blacks live in racially mixed regions their risk for being shot by the police is lower than when they live in segregated regions. Our results confirm the findings of Hattery and Smith (2007) that noted that Blacks living in the Deep South had better well-being outcomes when they lived in integrated counties compared to those living in segregated counties. As the population of officers that have fired their service weapon on the job is approximately 27% (Morin and Mercer 2017), the relationship between community geography and demography with risk for Blacks being shot by police is an affirmation of structural racism, regardless of individual level prejudice. We found that the dissimilarity metric used commonly in the literature is the least effective residential segregation metric in differentiating differences in police shootings ratings (Siegel et al. 2019; Gaynor et al. 2021). Instead, the strength and consistency of measures of interaction and ethnic diversity suggest that racialized behavior is driven more through engagement and presence than through evenness (or a lack thereof), perhaps mediated through a process of racial incongruence (Gaston et al. 2021). This differentiation suggests a much broader set of regions that would benefit from interventions.

Additionally, we investigated whether ecological drivers were more present in racially specific situations. Areas with high urbanization, poor housing security, and a lack of racial diversity have higher rates of Black victim police shootings. Areas with lower racial diversity have a much lower rate of police shootings of Hispanic victims. Hispanic communities have been found to have significantly different relationships with the police compared to Black Americans (Ong and Jenks 2004). Communities where individuals identify as White despite having Hispanic origins report higher community unity, have higher socioeconomic status, a better grasp of the English language, are native born, and are more likely to assimilate with the White population (South et al. 2005a, b). Our results may be evidence of a continued “Hispanic Paradox” (Franzini et al. 2001; Chenane and Wright 2021). The difference in effects between Blacks and Hispanics is frequently noted across the residential segregation literature, showing up in studies on breast cancer (Priutt et al. 2015), cardiometabolic risk (Mayne et al. 2019), in addition to those on policing (Holmes et al. 2018; Feldman et al. 2019; Zhao et al. 2019). These different outcomes from the same residential characteristics suggest that it is the interaction of location and race or ethnicity that modifies some element of police officer behavior (Mears et al. 2017). However, our results could also be a function of reporting bias in our underlying dataset. In particular, crowdsourced data may be challenged in differentiating between individuals who identify or appear as White but might have Hispanic origins.

There are some limitations that should be considered in the interpretation of this analysis. The magnitude of segregation in any given location is the outcome of complex processes taking place at multiple scales (Fowler 2018), and our chosen level of census tracts may suffer from an incomplete understanding of the spatial mechanisms of segregation. The dataset on police shootings provides no detail on the race or ethnicity of the police officer, which limits our ability to test the effect of racial or ethnic differences or officer–victim discordance with segregation on the shooting outcome. Results specific to fatal shootings by police might not be applicable to all uses of force, specially those that in non-fatal injuries. Finally, the information on arrest or policing rates beneath the county level is highly uneven.

Our results are also limited in degree of missingness of race and ethnicity information for the victim. It is possible that there is an overrepresentation of particular racial groups that are more likely to be mentioned, but it is not possible to determine the direction of bias. Analyzing this behavior at the tract level also has the effect of combining the behaviors of differentiated police forces, and different agencies could drive engagement behaviors (Shane et al. 2017). More precise data will be needed to identify the modifiable factors that will reduce racial and ethnic disparities in avoidable circumstances.

## Conclusions

Gun violence has evolved from a neighborhood problem into a social network problem that is a public health concern (Green et al. 2017). Recent studies have found similar results that show the likelihood of an event occurring which varies based on census tract social polarization (Feldman et al. 2019). We examined the differing effects that racial evenness, exposure, and population diversity have on the racially distinct outcome of police shootings. Hispanics protective effects were visible in regions with less evenly distributed Hispanic populations, but there was no effect when modeling focused on the likelihood of majority–minority encounters. Black populations were at higher risk for police shootings in regions with less even Hispanic or Asian distributions. All populations except Hispanics were more at risk in regions that were more diverse. Our modeling to connect residential segregation to regions with more than one fatal shooting did not observe effects substantially different from models examining a single shooting or any nonzero number.

We echo policy limitations elsewhere that efforts must be made in jurisdiction that oversee diverse and segregated communities to address not just the individual interactions police have with residents, but also of the frequency of these interactions and the locational context within which they are occurring (Siegel et al. 2021). We affirm the importance of indirect effects of disparities in socioeconomic status that can alter the police behavior in a region. Black communities often have poor access to health care, education, employment, and housing (Hahn et al. 2018), and police may be called to respond to evictions or illness that might be treated at a hospital more frequently in regions that lack non-criminogenic resources. In areas with segregated minority populations, the unequal amount of time spent may account for uneven outcomes in police encounters and shootings (Hattery and Smith 2021). With our results and the understanding that police shootings are shaped by a number of cultural and organizational factors (Prenzler et al. 2013), trust-building and de-escalation police training should be concentrated in police departments serving highly segregated communities (Diehr and McDaniel 2018, Engel et al. 2022). Our findings align with scholarship elsewhere showing that Black communities are more willing to engage in police partnerships, and concentrated disadvantage further enhances this receptiveness (Wehrman and De Angelis 2011). We believe that direct confrontation of these matters by criminologists and law enforcement of all levels will be necessary to stem the racist outcomes in police shootings that exist in American society.

## Data Availability

All data utilized in this analysis are publicly available from the US American Community Survey and Washington Post Fatal Force Database.

## Abbreviation

SVI: Social Vulnerability Index

## Footnotes

1 Consistent with the scholarly literature on race and policing, we use the terms "Black" and "White" to denote people’s racial identities as well as the demographic makeup of institutions (police departments) and communities or neighborhoods. We utilize capitalization throughout the document to emphasize the person-centric nature of these terms (nouns) as opposed to colors (adjectives). See the statement from the Associated Press. “The Associated Press style is now to capitalize Black in a racial, ethnic, or cultural sense, conveying an essential and shared sense of history, identity, and community among people who identify as Black… The lowercase black is a color, not a person." (AP 2020).
